# Enhanced angiogenic properties of umbilical cord blood primed by OP9 stromal cells ameliorates neurological deficits in cerebral infarction mouse model

**DOI:** 10.1038/s41598-023-27424-7

**Published:** 2023-01-06

**Authors:** Yasunori Yoshida, Yuki Takeda, Kenichi Yamahara, Hanae Yamamoto, Toshinori Takagi, Yoji Kuramoto, Akiko Nakano-Doi, Takayuki Nakagomi, Toshihiro Soma, Tomohiro Matsuyama, Nobutaka Doe, Shinichi Yoshimura

**Affiliations:** 1 Department of Neurosurgery, Hyogo Medical University, 1-1 Mukogawa, Nishinomiya, Hyogo 663-8501 Japan; 2 Laboratory of Molecular and Cellular Therapy, Institute for Advanced Medical Sciences, Hyogo Medical University, 1-1 Mukogawa, Nishinomiya, Hyogo 663-8501 Japan; 3 Laboratory of Neurogenesis and CNS Repair, Institute for Advanced Medical Sciences, Hyogo Medial University, 1-1 Mukogawa, Nishinomiya, Hyogo 663-8501 Japan; 4 Department of Occupational Therapy, School of Rehabilitation, Hyogo Medical University, 1-3-6 Minatojima, Chuo-Ku, Kobe, Hyogo 650-8530 Japan; 5 Department of Hematology, Hyogo Medical University, 1-1 Mukogawa, Nishinomiya, Hyogo 663-8501 Japan; 6 Department of Therapeutic Progress in Brain Diseases, Hyogo Medical University, 1-1 Mukogawa, Nishinomiya, Hyogo 663-8501 Japan

**Keywords:** Translational research, Stroke

## Abstract

Umbilical cord blood (UCB) transplantation shows proangiogenic effects and contributes to symptom amelioration in animal models of cerebral infarction. However, the effect of specific cell types within a heterogeneous UCB population are still controversial. OP9 is a stromal cell line used as feeder cells to promote the hematoendothelial differentiation of embryonic stem cells. Hence, we investigated the changes in angiogenic properties, underlying mechanisms, and impact on behavioral deficiencies caused by cerebral infarction in UCB co-cultured with OP9 for up to 24 h. In the network formation assay, only OP9 pre-conditioned UCB formed network structures. Single-cell RNA sequencing and flow cytometry analysis showed a prominent phenotypic shift toward M2 in the monocytic fraction of OP9 pre-conditioned UCB. Further, OP9 pre-conditioned UCB transplantation in mice models of cerebral infarction facilitated angiogenesis in the peri-infarct lesions and ameliorated the associated symptoms. In this study, we developed a strong, fast, and feasible method to augment the M2, tissue-protecting, pro-angiogenic features of UCB using OP9. The ameliorative effect of OP9-pre-conditioned UCB in vivo could be partly due to promotion of innate angiogenesis in peri-infarct lesions.

## Introduction

Cerebral infarction is one of the major causes of permanent impairment and death. Currently, intravenous thrombolytic agents and endovascular recanalization therapy are widely employed in the acute phase to prevent ischemic penumbra. However, these therapies have narrow time windows and therapeutic indications are limited to a specific patient population^1^. Therefore, therapeutic innovation for cerebral infarction is necessary and highly demanding.

Compared with conventional reperfusion therapies, cell therapy is recently expected to be a more widely applicable therapy for cerebral infarction in future^2^, because it has the potential to ameliorate the associated symptoms even when it is administered during the subacute or chronic phases^3^.

Previous studies have suggested that umbilical cord blood (UCB) and adult peripheral blood (PB) contain a certain mononuclear fraction that can accumulate at the sites of active angiogenesis and contribute to neovascularization^4,5^. Asahara et al. first described that CD34^+^ hematopoietic cell population sorted from human PB-derived mononuclear cells (PB-MNCs) could yield spindle-shaped cells with the universal hematopoietic marker CD45 by plating onto fibronectin-coated dishes, and could promote neovascularization in a hind limb ischemia model^6^. Similar results were reported by Kalka et al. using primary adherent mononuclear cells without CD34 sorting^7^.

Since then, the pro-angiogenic cell population within the heterogeneous population of mononuclear cell fraction has been investigated extensively. CD14^+^CD45^+^ monocytic cells in CD34^+^CD45^+^ hematopoietic progenitors in UCB or PB have been shown to form cell clusters similar to those reported by Asahara^8^, whereas some other studies reported that the CD34^-^CD14^+^ monocytic fraction of PB had similar features^9,10^. Meanwhile, a population of pro-angiogenic cells has been shown to originate from myeloid progenitors and granulocyte–macrophage progenitors^11^. These findings are in agreement with the well-known notion that monocytes have a range of different functional phenotypes (i.e., M1 and M2) and that M2 monocytes are associated with angiogenesis^12^ as well as promotion of neurogenesis, axonal sprouting, and remyelination^13^, whereas M1 monocytes are pro-inflammatory and mediate tissue damage.

In fact, such cell fractions have been shown to facilitate revascularization of ischemic tissues such as ischemic hind limb and in myocardial infarction^14,15^. Furthermore, angiogenesis is known to be positively correlated with neurological recovery after cerebral infarction^16^. Several studies demonstrated that UCB transplantation showed pro-angiogenic effects and contributed to a reduction in the ischemic volume or symptom amelioration in animal models of cerebral infarction^17–19^. However, most of these studies used crude UCB^17,19,20^ or UCB-derived mononuclear cells^18,21^ similar to those reported by Kalka et al. Moreover, the efficacy of CD34^+^ cells sorted from UCB-derived mononuclear cells^6^ in a model of cerebral infarction is controversial^21,22^.

These findings led us to hypothesize that augmentation of more specific pro-angiogenic cell populations in UCB leads to enhanced therapeutic effects. Based on this hypothesis, we focused on the features of OP9, a stromal cell line, that was initially used as feeder cell line to promote erythroid, myeloid, and lymphoid differentiation of embryonic stem cells^23^. It is also known to facilitate differentiation into endothelial and hematopoietic cells from a cell population derived from the pre-liver intraembryonic hematopoietic site^24^. Additionally, a certain endothelial progenitor cell population derived from the inner surface of blood vessels was recently reported in several organs. OP9 effectively promoted their expansion, colony formation, and differentiation into mature endothelial cells^25^. Thus, OP9 might be suitable for enhancing the angiogenic properties of UCB.

In this study, we established a new culture method for UCB cells primed with OP9. We investigated whether this ‘pre-conditioning’ method of UCB could enhance its angiogenic properties, and to identify the underlying mechanisms and the impact of OP9 pre-conditioned UCB on behavioral deficiencies caused by an experimental focal cerebral infarction in a mouse model using surgical occlusion of the middle cerebral artery (MCAO).

## Result

### OP9 pre-conditioning facilitated network formations of UCB cells in Matrigel

**Figure 1:**
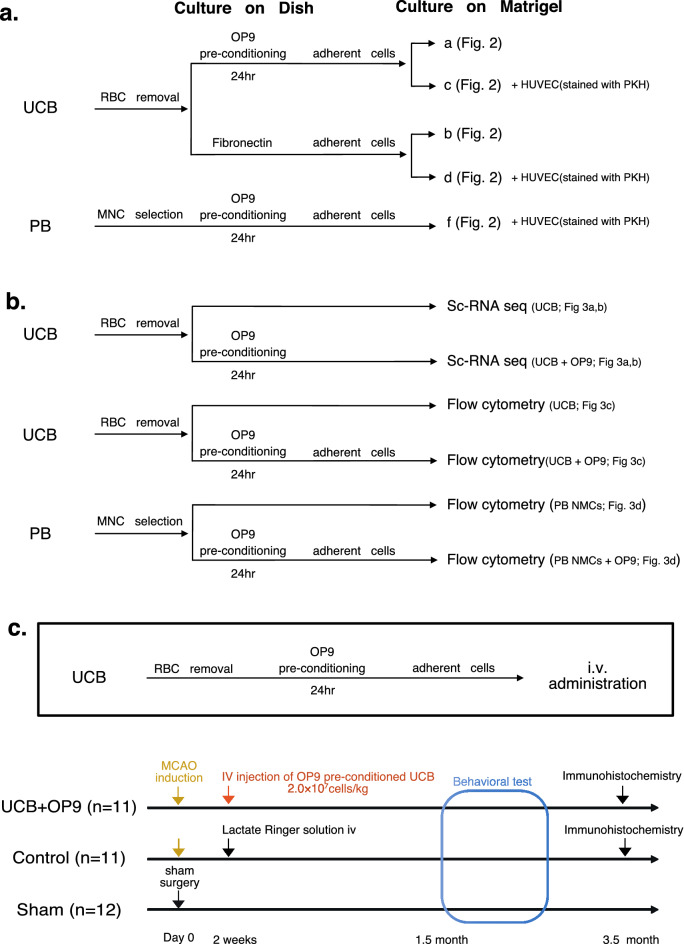
Methodological schematic representations for the study. (**a**) Protocol for the network formation assay. RBC depleted human umbilical cord blood (UCB) cells and peripheral blood-derived mononuclear cells (PB-MNCs) were co-cultured on OP9 stromal cells (i.e. OP9 pre-conditioning) in 10-cm dishes. After 18–24 h culture, adherent cells were harvested and subjected to in vitro network formation assay. RBC depleted crude UCB cells and PB-MNCs cultured on fibronectin-coated dishes were used as control. (**b**) Protocol for the single cell RNA sequencing (scRNA-seq) and flow cytometry. RBC depleted crude UCB cells were used as control. (**c**) Protocol for intravenous administration of OP9 pre-conditioned UCB in the MCAO mouse model. Mice were randomly assigned to three groups as follows: the UCB + OP9 group (n = 11); the control group (n = 11); and the sham surgery group (n = 12) and underwent MCAO or sham surgery. Two weeks after surgery, mice in the UCB + OP9 group received 100 μL of OP9 pre-conditioned UCB (2.0 × 10^7^ cells/kg) and mice in the control group received the same amount of lactated Ringer’s solution intravenously (iv.) Next one month after cell transplantation, a series of behavioral tests were performed, and three months after cell transplantation, five mice in the UCB + OP9 group and five mice in the control group were randomly selected and subjected to the immunohistochemistry.

**Figure 2:**
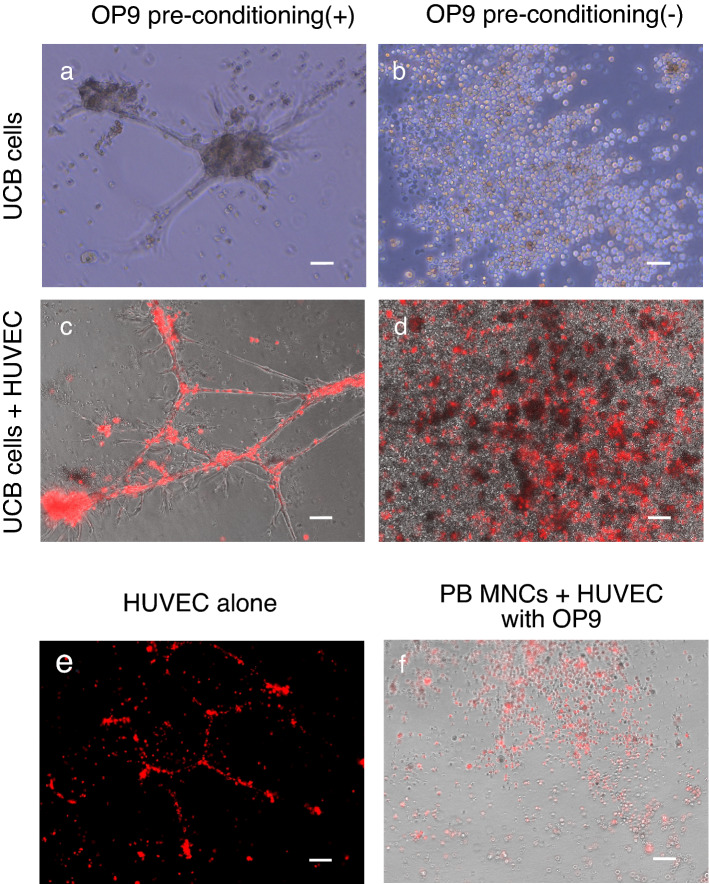
In vitro network formation assay using UCB- or PB-MNC-derived adherent cells with or without OP9 pre-conditioning. The panel names of (**a**–**f**) are identical to those of (**a**–**f**) in Fig. 1a. UCB- or PB-MNC-derived adherent cells with or without OP9 pre-conditioning were seeded onto Matrigel. Human umbilical vein endothelial cells (HUVECs) were added in panels (**c**)–(**f**). (**a**) OP9-pre-conditioned UCB cells showing capillary-like structures. (**b**) UCB cells without OP9 pre-conditioning. (**c**) OP9-pre-conditioned UCB cells with HUVECs. (**d**) HUVECs and UCB cells without OP9 pre-conditioning. (**e**) HUVECs alone (without OP9 pre-conditioning). (**f**) OP9-pre-conditioned PB-MNCs co-cultured with HUVECs. Scale bar: 100 μm, 10 × magnification.

In vitro network formation assays on Matrigel have long been used to assess the pro-angiogenic capacity of cultured cells^5,8,10,26–29^. To assess the pro-angiogenic characteristics of UCB cells, we performed the assay using PB-MNCs, UCB cells, and OP9 pre-conditioned UCB cells (Fig. 1a). Surprisingly, following a short co-culture within 24 h, OP9-pre-conditioned UCB cells formed capillary-like structures, whereas such structures were not observed in UCB cells without pre-conditioning (Fig. 2a,b). When incubated with human umbilical vein endothelial cells (HUVECs), OP9 pre-conditioned UCB cells formed a thick and solid network structures heterogeneously aligned with HUVECs, whereas UCB cells without OP9 pre-conditioning failed to form such structural alignment with HUVECs (Fig. 2c,d). Compared with OP9-pre-conditioned UCB cells, HUVECs also formed a thin and weak network structure on their owns (Fig. 2e). PB-MNCs were used as controls, which failed to form capillary-like structures with HUVECs even after OP9 pre-conditioning (Fig. 2f). OP9 cells alone did not form any capillary-like structures (data not shown).

Therefore, OP9 pre-conditioning augmented the pro-angiogenic characteristics of UCB cells within 24 h, which were not induced in PB-MNCs.

### OP9 pre-conditioning shifted the phenotype of the monocytic fraction in UCB cells from M1 to M2-dominant status

UCB cells contain heterogeneous cell populations, hence it is necessary to clarify the cell types affected by OP9 pre-conditioning. Single-cell RNA sequencing (scRNA-seq) was used to evaluate changes in the genetic expression profile of each cell population, where we compared both OP9 pre-conditioned and crude UCB cells (Supplementary Fig. 1). Unsupervised clustering using t-distributed stochastic neighbor embedding (tSNE) was employed to organize all UCB cells into transcriptionally distinct clusters and identify the differentially expressed marker genes in each cluster. A total of 25 stable clusters emerged, including clusters of monocytes, granulocytes, hematopoietic stem cells, and common myeloid and granulocyte–macrophage progenitors (CMP/GMPs), which are known to have pro-angiogenic effects (Supplementary Fig. 1). A list of the genes preferentially expressed in each cell type is provided in the supplementary data. A violin plot and gene expression distribution map of CD14 and CGR3A, genes considered to be expressed preferentially in monocytes, suggested that cells in cluster 4 were mainly composed of monocytes (Supplementary Fig. 1). The monocytic fraction was considerably higher in OP9 pre-conditioned UCB cells than that in the crude UCB cells (8.0% vs. 5.3%), whereas the cell count of hematopoietic stem cells and CMP/GMPs did not change (0.6% vs. 1.1% and 0.6% vs. 0.4%, respectively) (Supplementary Fig. 1).

**Figure 3:**
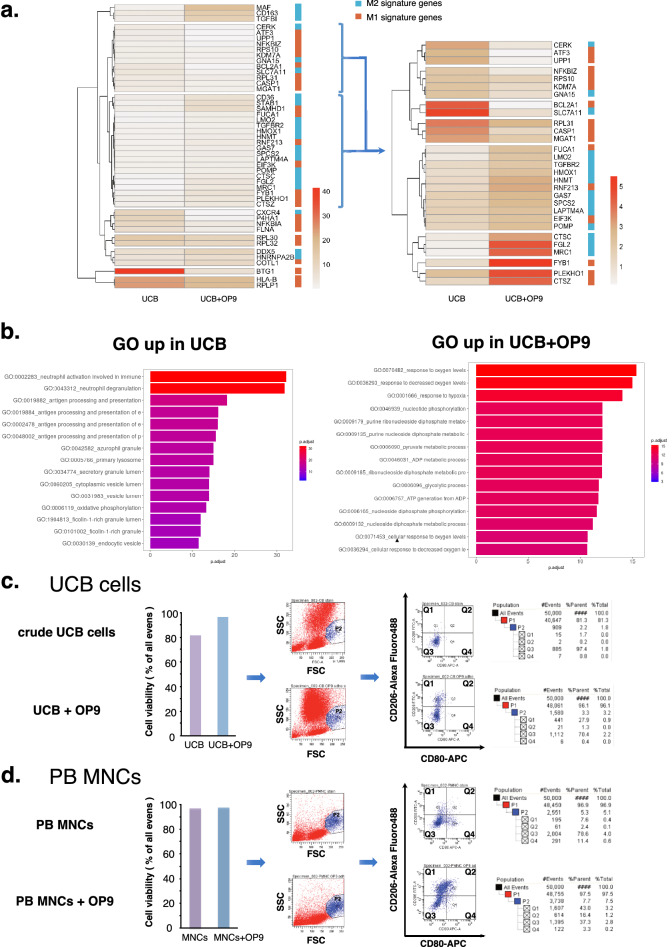
Single-cell RNA sequencing (scRNA-seq) and flow cytometry analysis of crude UCB and OP9 pre-conditioned UCB cells. (**a**) Heatmaps of typical M1 and M2 signature genes. Right sidebar depicts M1 and M2 signature genes in blue and red respectively. Scale bar indicates gene expression levels calculated from log normalized unique molecular index (UMI) counts (scale factor = 10,000). (**b**) Gene ontology (GO) enrichment analysis of differentially expressed genes between crude UCB and UCB + OP9. Enriched GO terms were identified based on the hypergeometric test and adjusted *p* values were derived by the Benjamini and Hochberg (BH) method. Top 15 enriched GO terms were shown according to –log10 *p* value. (**c**, **d**) Flow cytometry analysis of UCB and UCB + OP9. Cell viability (the ratio of living cells to all events) was analyzed using DAPI staining. Cells in monocytic fraction (P1 population) were gated based on forward angle light scatter (FSC) and side angle light scatter (SSC) characteristics. Then, CD80 and CD206 were used to sort M1 and M2 type cells into Q4 and Q1 quadrants, respectively.

M1 phenotypic monocytes are pro-inflammatory and mediates tissue damage, whereas the M2 phenotype is associated with angiogenesis. Hence, we focused on the gene expression features of the monocytic cluster. The heatmaps of typical M1 and M2 signature genes showed a clear tendency of the gene expression profile, where the M2 marker genes, such as macrophage activating factor (MAF) and CD163, were expressed abundantly in the monocytic cluster from OP9 pre-conditioned UCB, while the M1 specific gene expression was higher in that from crude UCB (Fig. 3a). These findings suggest that the monocytic cluster from OP9 pre-conditioned UCB was shifted toward the M2 phenotype compared to that from crude UCB.

Additionally, gene ontology (GO) analysis of differentially expressed genes between the monocytic cluster of OP9 pre-conditioned and crude UCB revealed characteristic trends of GO term enrichment. GO terms related to hypoxia, glycolysis, and oxidative pathways had the highest enrichment *p *values (Fig. 3b). These findings are in concordance with the results of heatmaps in terms of intracellular metabolic changes, considering that hypoxia shifts monocytes toward the M1 phenotype in which energy is obtained mainly through glycolysis, whereas that in M2 monocytes through oxidative pathways^30^. To further investigate the pro-angiogenic mechanisms in monocytic fraction, we compared the gene expression profiles associated with the regulation of cell migration involved in sprouting angiogenesis (GO:0090049) in monocytic clusters of OP9-pre-conditiond UCB and crude UCB. The genes were broadly up-regulated in the monocytic cluster of OP9-pre-conditioned UCB (Table 1). Among these genes, the expression of neuropilin-1 (Nrp1) was enhanced in OP9-pre-conditioned monocytic fraction compared with that in crude UCB. Nrp1 is a transmembrane receptor binding with VEGF_165_ and class 3 semaphorin and known to be expressed in a certain pro-angiogenic population of monocyte (i.e. neuropilin-1 expressing monocyte; NEM)^31^. Therefore, pro-angiogenic mechanisms of OP9-pre-conditioned monocytic fraction could be partially explained by augmentation of NEM by OP9 pre-conditioning.

**Table 1:** Gene expression profiles associated with angiogenesis regulation in the monocytic cluster.

Up-regulated in UCB + OP9			Up-regulated in crude UCB		
Gene	Fold change	p	Gene	Fold change	p
Hmox1	2.2	< .01	Hdac5	0.42	< .01
Itgb1bp1	3.1	< .01	Thbs1	0.69	< .01
Map3k3	2.4	< .01	Vegfa	0.057	< .01
Nrp1	1.8	< .01			
Pdcd10	1.6	< .01			
Rhoa	1.3	< .01			
Spred1	5.3	< .01			
Cib1	1.5	< .01			

To reinforce the results of scRNA-seq, we investigated the M1/M2 phenotypic shift of these two monocytic fractions using flow cytometry analysis. First, we assessed the viability of UCB cells using 4′,6-diamino-2-phenylindole (DAPI) staining, which revealed a higher viability of UCB cells in OP9 pre-conditioned UCB than that in crude UCB (96.1% vs. 81.3% of all events; Fig. 3c), suggesting the protective effect of OP9. CD80 and CD206 were used to differentiate M1 and M2 monocytes^32,33^. The percentage of CD80^-^CD206^+^ M2 type cell count was much higher in the monocytic fraction from OP9 pre-conditioned UCB compared with that from crude UCB (27.9% vs. 1.7%; Q1 quadrant in Fig. 3c), while the percentage of the total monocytic cells was comparable in both OP9 pre-conditioned and crude UCB-derived cells (3.3% vs. 2.2% of all living cells; P2 population in Fig. 3c).

Additionally, we also observed a prominent tendency of M1-M2 shift in PB-MNCs owing to OP9 pre-conditioning. The percentage of CD80^−^ CD206^+^ cells was substantially higher (43.0% vs. 7.6%; Q1 quadrant in Fig. 3d), while that of CD80^+^ CD206^−^ cells was lower (3.3% vs. 11.4%; Q4 quadrant in Fig. 3d) in the monocytic fraction from OP9-pre-conditioned PB-MNCs compared with that from crude PB-MNCs (Fig. 3d).

In several granulocytic clusters of scRNA-seq, the fraction of granulocytes was higher in OP9-pre-conditioned UCB cells than in the crude UCB cells (granulocyte_3,4, and _5, supplementary Fig. 1). Similar to monocytes, neutrophils are also known to have N2 phenotype that induces angiogenesis^34^. Therefore, we performed the same flow cytometry analysis in granulocytic fraction (P2 population gated by SSC and FSC characteristics in supplementary Fig. 2). However, CD80^-^CD206^+^ N2 type cells were only found in 0.12% of granulocytic fraction in OP9 pre-conditioned UCB (Q1 quadrant in supplementary Fig. 2).

Overall, the 24 h short time OP9 pre-conditioning strongly shifted the phenotypic characteristics of the monocytic fraction in UCB cells and PB-MNCs toward M2 dominant state, as well as its protective effect in terms of the UCB cell viability.

### Intravenous administration of OP9 pre-conditioned UCB promoted angiogenesis in peri-infarct lesions of MCAO mice

**Figure 4:**
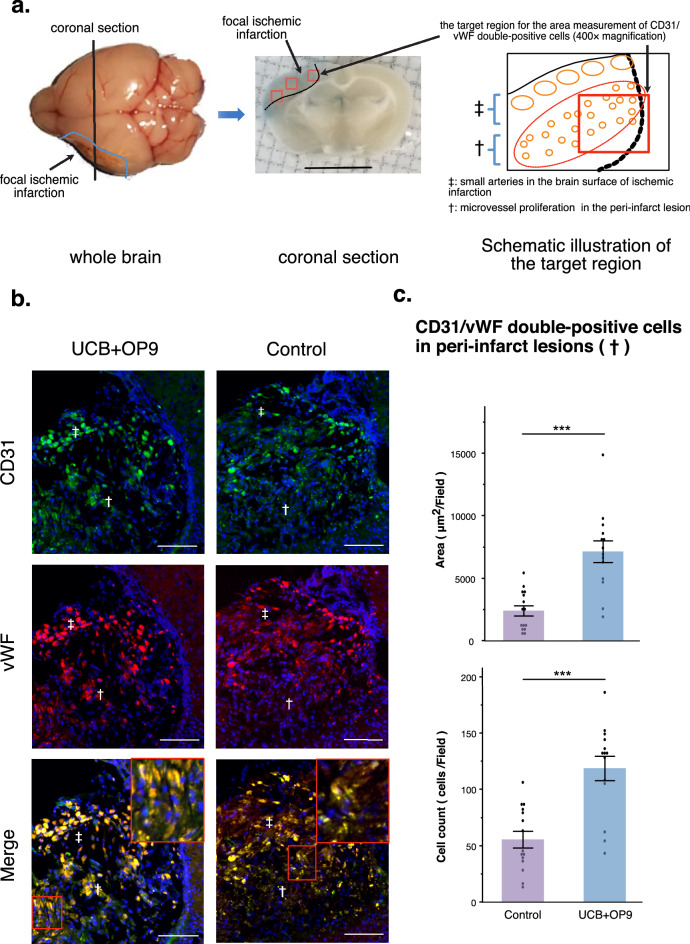
vWF and CD31 staining in the peri-infarct lesion of MCAO mice with or without OP9-pre-conditioned UCB administration. Fluorescent immunohistochemistry was performed 3.5 months after surgery (n = 5 in the UCB + OP9 group and n = 5 in the control group, respectively). (**a**) Schematic illustration of the target region for the area measurement of CD31/vWF (von Willebrand factor) double-positive cells by a fluorescence microscope. One coronal section per mouse (n = 5 in each group) was analyzed. Small arteries in the brain surface of ischemic infarction (‡) and microvessels in the peri-infarct lesion located at the periphery of the ischemic infarction adjacent to the normal cortex and striatum (†) were observed. Three non-overlapping random visual fields of (†) indicated by red solid boxes (400 × magnification) per coronal section were analyzed to measure the CD31/vWF double-positive area. (**b**) Representative images of immunofluorescent staining of brain sections. CD31/vWF double positive cells (yellow) with CD31^+^ (green) and vWF^+^ (red) in the peri-infarct lesions (†) were subjected to the analysis in (**c**). Nuclei were counterstained using DAPI (blue). Scale bar: 50 μm. 200 × magnification. (**c**) The area measurement and cell count of CD31^+^/vWF^+^ cells per field observed in 400 × magnification (n = 15 per each group). ***p < 0.001, unpaired t-test.

The pro-angiogenic effect of OP9 pre-conditioned UCB cells was assessed by intravenous administration in the MCAO mouse model. The repair process, prominently angiogenesis, occurs in the subacute phase after cerebral infarction^35,36^. Hence, we administered OP9 pre-conditioned UCB cells 2 weeks after MCAO induction to enhance the angiogenic process and sacrificed these MCAO mice 3 months after UCB administration (Fig. 1b), considering that the density of microvessels in the peri-infarct lesion was reported to peak by day 30 and gradually decrease in 3 months^37^. The morphological observations in the focal ischemic infarction were similar to that reported by the previous report^38^, with small arteries in the brain surface of ischemic infarction, and microvessels in the peri-infarct lesion located at the periphery of the ischemic infarction adjacent to the normal cortex and striatum (Fig. 4a). We assessed the density of microvessels in the peri-infarct lesion by the area measurement and cell counting of CD31^+^/vWF^+^ (von Willebrand factor) cells. The CD31/vWF double-positive area was significantly higher in the OP9 pre-conditioned UCB-administered group (n = 5) than that in the vehicle control (n = 5) (***p < 0.001; Fig. 4b,c). This result suggests that OP9 pre-conditioned UCB cells had prominent pro-angiogenic effects on the peri-infarct lesion of MCAO mice compared to crude UCB cells.

### Intravenous administration of OP9 pre-conditioned UCB ameliorated neurobehavioral abnormalities in MCAO mice

Finally, we performed behavioral tests in MCAO mice to assess the effects of OP9 pre-conditioned UCB on improvement of neurological disturbances caused by cerebral infarction. Behavioral tests were conducted 1.5 month after surgery and compared within three groups [the UCB + OP9 group (n = 11), the control group (n = 11), and the sham surgery group (n = 12)], as shown in Fig. 1c.

Before multiple comparisons, a one-way analysis of variance (ANOVA) for the Y-maze task was performed, where it showed significance [F(2) = 19.35, *p* < 0.0001]. Further, a two-way repeated measures ANOVA (rmANOVA) (i.e., the open field test, the passive avoidance learning task, and the forced swimming test) revealed significance in the groups in all tasks [F(2, 32) = 10.60, *p* < 0.0003; F(2, 32) = 6.08, *p* = 0.0058; F(2, 32), *p* < 0.0001, respectively], as well as in the time effect of the passive avoidance learning task [F(2, 31) = 14.48, *p* < 0.0001] and the group by time interaction in the forced swimming test [F(10, 160) = 3.179, *p* = 0.001]. However, rmANOVA in the wire hung test failed to show significance.

**Figure 5:**
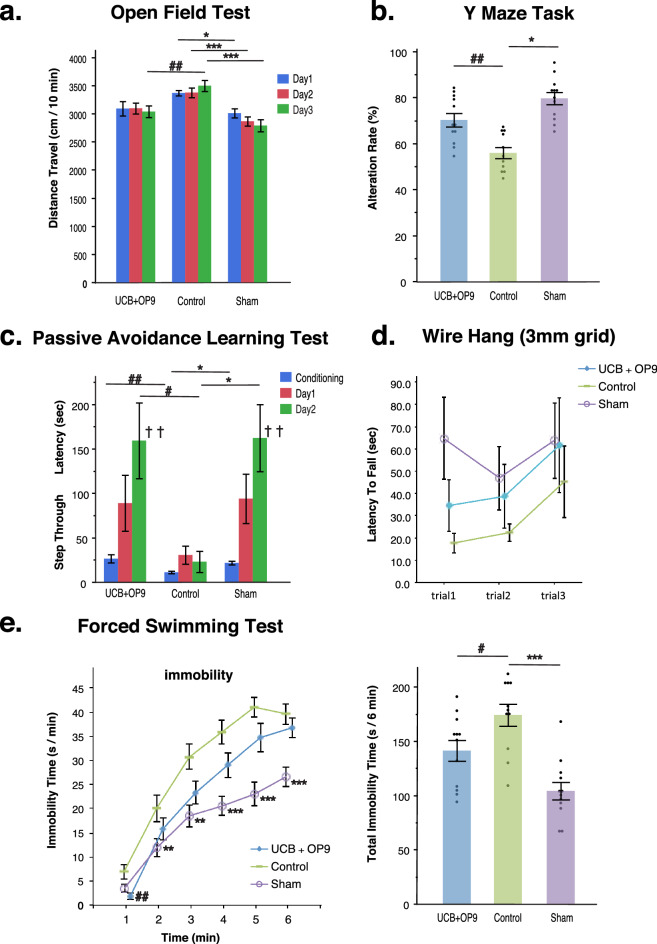
Behavioral tests for MCAO mice with or without OP9-pre-conditioned UCB administration. Mice were randomly assigned to three groups: the UCB + OP9 group (n = 11), the control group (n = 11), and the sham surgery group (n = 12). The open field test (**a**), the Y-maze task (**b**), the passive avoidance learning test (**c**), the wire hang test (**d**), and the forced swimming test were conducted 1.5 month after the surgery. Before multiple comparisons, one-way analysis of variance for the Y-maze task and a two-way repeated measured ANOVA for data arising from repeated measures (i.e., the open field test, passive avoidance learning task, wire hang test, and the forced swimming test) were used. A; *p* < 0.05, Dunnett’s test for differences between-subject. *p < 0.05, **p < 0.01, ***p < 0.001, Dunnett’s test in comparison of the sham surgery group with the control group (i.e., the MCAO induced group). #*p* < 0.05, ##*p* < 0.01, Dunnett’s test in comparison of the OP9 + UCB group (i.e., the MCAO induced and OP9-pre-conditioned UCB-administered group) with the control group. ^†^*p* < 0.05, ^††^*p* < 0.01, Dunnett’s test compared with the conditioning trials of each group.

For multiple comparisons, first, we compared the control and sham-surgery groups to assess whether MCAO induction affected the score of each behavioral test, and whether these tests were suitable for detecting abnormal neurological functions caused by MCAO. In the open field test, travel distance in the control group were significantly higher than those in the sham surgery group throughout the experimental duration, reflecting an abnormal lack of habituation and anxiety in the control group (*p < 0.05, **p < 0.01, ***p < 0.001; Fig. 5a). Although the time effect in rmANOVA was not statistically significant, a trend toward a time-dependent decline in travel distance was observed in the sham surgery group, indicating a normal habituation reaction in the open field test. In the Y-maze task, the alternation rate significantly decreased in the control group compared to that in the sham surgery group, which suggested an impairment in working memory in the control group (*p < 0.05; Fig. 5b). Further, in the passive avoidance learning task, the step-through latency was elongated in the sham surgery group after every trial day, while that in the control group did not change, indicating that mice in the sham surgery group successfully acquired avoidance behavior, but mice in the control group did not, which may be attributed to nociceptive memory impairment (^††^*p* < 0.01, *p < 0.05, Fig. 5c). The forced swimming test showed that mice in the sham surgery group had a shorter immobility time than mice in the control group, indicating the development of a loss of motivation and impairment of exercise tolerance in MCAO mice (**p < 0.01, ***p < 0.001; Fig. 5e).

Next, we compared the control and OP9 + UCB groups to investigate the ameliorative effects of OP9 pre-conditioned UCB cells on behavioral task scores. The scores on the open field test, Y-maze test, passive avoidance learning task, and forced swimming test were significantly better in the OP9 + UCB group than those in the control group (^#^*p* < 0.05, ^##^*p* < 0.01, ^††^*p* < 0.01). In the wire hang test, we could not detect significant differences among the three groups (Fig. 5d), because our MCAO model is known to show rapid recovery from focal motor deficits and is not always sufficient for detecting ameliorative effects on motor function^39,40^. However, there was a trend that the latency to fall was shortened by MCAO induction (i.e., shorter in the control group than in the sham surgery group) and improved by the administration of OP9-pre-conditioned UCB cells (i.e., longer in the OP9 + CUB group than in the control group), suggesting the ameliorative effect of OP9-pre-conditioned UCB cells in muscle weakness and exercise tolerance. Therefore, these results indicate that the behavioral abnormalities caused by MCAO, such as general activity, memory impairment, exercise tolerance, and depression-like symptoms, were improved by the administration of OP9 pre-conditioned UCB cells.

## Discussion

The current study demonstrated that OP9 strongly and rapidly augmented the pro-angiogenic characteristics of UCB cells (Fig. 2), partly by shifting their phenotype in the monocytic fraction toward an angiogenic M2 dominant status (Fig. 3). It was also demonstrated that subacute intravenous administration of OP9 pre-conditioned UCB cells ameliorated neurological deficits in the MCAO mouse model (Fig. 5), partly due to promotion of innate angiogenesis in peri-infarct lesions (Fig. 4). To the best of our knowledge, this is the first study to report a strong, easy, and fast modification technique for UCB to augment its pro-angiogenic features and verify its biological effects in a subacute cerebral infarction model.

Asahara et al. first described the nomenclature of endothelial progenitor cells (EPC), a circulating cell population that can contribute to neovascularization by accumulating in the sites of active angiogenesis^6^. In in vitro cell culture assay, two different EPC-populations are reported: early and late EPCs, the former also called as myeloid angiogenic cells (MACs) or pro-angiogenic hematopoietic cells, whereas the latter is also called as endothelial colony forming cells (ECFCs) or endothelial outgrowth cells^41,42^. They were both adherent cells isolated from PB-MNCs plated onto fibronectin or collagen-coated dishes under endothelial cell culture conditions but differed in their time of arising in culture^26,29,43,44^. Originally, it was reported that early EPC was a spindle shaped cell population emerging at 2–3 weeks and ceased at 4 weeks, whereas late EPC was a cobblestone shaped cell population emerging at 2–3 weeks, grew exponentially at 4–8 weeks and had capacity of multiple population doublings without senescence^27^. ECFCs have specific endothelial progenitor characteristics such as significant proliferative capacity, network-formation potential in Matrigel and an ability to contribute to de novo blood vessel formation in vivo^26,29,45^. In contrast, MACs share none of the properties of ECFCs, but have hematopoietic-derived monocytic features (i.e., expression of myeloid progenitor cell markers and the ability to differentiate into macrophages), and promote angiogenesis through a paracrine mechanism^12,43–46^.

Based on previous studies, MACs contain a monocyte fraction and possess similar characteristics with cell populations widely used for cell therapies to promote angiogenesis^14^. Our OP9 pre-conditioned UCB cells also contain monocyte fraction which possesses the characteristics of MACs, such as pro-angiogenic effects through a paracrine mechanism. However, in the network formation assay, OP9 pre-conditioned UCB cells formed a network structure and heterogeneously aligned with HUVECs, whereas PB-MNCs did not form a network structure, although both PB-MNCs and UCB cells shifted toward the M2 phenotype by using OP9 pre-conditioning. Therefore, it seems to be appropriate to interpret our findings in the network formation assay as follows: (i) our OP9 pre-conditioning shifted the monocyte fraction in UCB cells toward the M2 phenotype and enhanced their pro-angiogenic effects, (ii) these monocytes did not differentiate into endothelial cells, and (iii) OP9 pre-conditioning might promote another undefined population in UCB cells to participate and align in the core structure of network heterogeneously with HUVECs, supported by surrounding M2-shifted pro-angiogenic monocytes. Previous reports demonstrated that MACs and ECFCs had a synergistic effect on neovascularization^43^ and unfractionated UCB-derived mononuclear cells were superior to CD34^+^ or CD34^-^ cell fractions in terms of their effects on the reduction of infarction volume and amelioration of neurological deficits^21^. Therefore, these findings suggest that our method using unfractionated UCB cells co-cultured with OP9 would be a better approach to augment angiogenic features, in which several innate cell populations related to angiogenesis were contained and modulated simultaneously by OP9 without isolating a specific cell population.

Among the various cell sources, UCB has some advantages in clinical application as a donor source of cell therapy, especially when aiming at neovascularization. First, UCB contains stem cells with a high proliferation potential^47^. Second, UCB contains a higher amount of pro-angiogenic cell populations than PB or bone marrow^48,49^. Third, UCB transplantation has a long history of being employed in clinics^50^, and has a lower incidence and severity of graft-versus-host disease than bone marrow transplantation^51,52^. Finally, UCB is obtained less invasively and easily because it is usually discarded after birth, and a stable supply system has been established. These characteristics further support our approach for the use and modulation of UCB in cell therapy.

Several studies have shown that M2 macrophages can be generated by co-culture with mesenchymal stem cells (MSCs)^53–55^. Furthermore, MSC-derived supernatants could potentiate macrophages toward an anti-inflammatory phenotype, and the administration of such “educated macrophages” significantly improved the healing process of tendon injury, reduced the endogenous M1/M2 macrophage ratio, and promoted angiogenesis^56^.

OP9 has been widely known as a feeder cell to promote hematoendothelial differentiation, but is also known to share some characteristic features with MSCs, including the immuno-phenotype, the ability of differentiation, and immunomodulative effects^57^. The underlying mechanisms of OP9 pre-conditioning in UCB cells could be attributed through “monocyte education” rather than differentiation of stem and progenitor cells, considering that we co-cultured UCB with OP9 for only 1 d while usually it takes days to obtain myelomonocytic cells through differentiation from embryonic stem cells using OP9^58^.

Although the underlying pro-angiogenic molecular mechanisms of in vivo M2-shifted monocytes were not clarified, an interesting insight was obtained from the expression profile of the monocytic fraction in scRNA-seq. The expression of Nrp1 was enhanced in the OP9-pre-conditioned monocytic fraction compared with that in crude UCB. Recently, several studies have demonstrated that (i) a certain population of monocytes expressed Nrp1 as a transmembrane receptor (i.e., neuropilin-1 expressing monocyte; NEM), (ii) Nrp1 bound with VEGF_165_ and class 3 semaphorin, and mediated chemo-attraction of NEMs toward the site of neoangiogenesis, and (iii) NEMs could recruit smooth muscle cells, promote vessel maturation in arterial formation, and reduce abnormal vascular permeability, although NEMs themselves were not incorporated into the vessel structure^31,59–61^.

The current study had certain limitations. First, we did not evaluate the difference in the effect on ischemic stroke between OP9 pre-conditioned and crude UCB. Second, although it is beyond the scope of this manuscript, it is necessary to assess the effect of OP9 on cell populations other than the monocytic fraction, such as UCB-derived AC133^+^ progenitor cells^5,62^ or UCB-derived ECFC^26^, as well as T cell subsets, which have been reported to hinder neurological recovery after stroke^21,63^. In this regard, further analysis of gene expression profiles using scRNA-seq may provide clues to answer these questions. Third, we did not verifiy the engraftment of administered UCB in the brain, nor investigated whether these UCB-derived cells differentiated and participated in the structure of repaired tissues. Several reports have demonstrated engraftment of UCB-derived cells^18,20,62,64,65^, while some reports failed^21,22^. Also, their differentiation ability is controversial. The discrepancies in these findings are partially due to differences in the timeline of UCB administration after infarction (24 h–1 week after MCAO), that of histological analysis (1 week–1 month) and the method of detecting human-derived UCB cells. Nonetheless, the mechanisms of symptom amelioration in the current study can be explained partly by the pro-angiogenic effect of OP9-pre-conditioned UCB cells on the innate tissue-repairing process, which has been reported previously^17,19,62^.

Although these limitations exist, our OP9 pre-conditioning method is still outstanding in terms of its fast, convenient, and feasible features, as well as its strong effect on modulating the bioactivity of UCB toward M2, a pro-angiogenic, tissue-protective phenotype.

## Conclusion

In this study, we found a strong and rapid method to augment the M2, pro-angiogenic, tissue-protective features of UCB by co-culturing with OP9. In addition, we demonstrated that subacute administration of OP9 pre-conditioned UCB ameliorated the behavioral deficiencies induced by MCAO in a mouse model, partly by promoting innate angiogenesis in peri-infarct lesions.

## Methods

### Cell preparation and culture

Written informed consent was obtained from all donors who provided UCB or PB, and the study protocol was approved by the Ethics Committee of Hyogo Medical University (approval number 0325) and performed in accordance with the Declaration of Helsinki.

OP9 cells were obtained from Dr. Nobuyuki Takakura (Department of Signal Transduction, Research Institute for Microbial Diseases, Osaka University, Japan) and maintained in α Minimum Essential Medium with nucleosides (Thermo Fisher Scientific, 41061-029; Waltham, MA) supplemented with 20% fetal bovine serum (Biowest, S1820; Nuaille, France) and penicillin–streptomycin (Sigma-Aldrich, P7539; St. Louis, MO, USA).

Human umbilical cord-derived HUVECs were purchased from Lonza (C2517A; Basel, Switzerland) and maintained in EGM-2 medium (CC-3162; Lonza).

RBC-depleted UCB and PB-MNCs were obtained using the EasySep RBC Depletion Reagent (Stem Cell Technologies, Vancouver, Canada) and Ficoll-Paque PLUS (Cytiva, 17144002, MA) according to the manufacturer’s protocol. The cells were resuspended in DMEM/F12 (Thermo Fisher Scientific, 11330-032) supplemented with epidermal growth factor (Peprotech, AF100-15; Cranbury, NJ), fibroblast growth factor basic (Peprotech, 100-18B), N-2 (Thermo Fisher Scientific, 17502048), and antibiotic–antimycotic (Thermo Fisher Scientific, 15240-062) and co-cultured on OP9 stromal cells (i.e., OP9 pre-conditioning) in 10-cm culture dishes at a density of 1 × 10^6^ cells/cm^2^. After 18–24 h of culture, non-adherent cells were removed, and adherent cells were harvested by trypsinization, washed once with medium, and then subjected to in vitro network formation assay, flow cytometry analysis, and intravenous administration in the mouse model. For scRNA-seq, a combination of adherent and supernatant cells derived from RBC-depleted UCB was used after OP9 pre-conditioning. Adherent cells obtained from RBC removed crude UCB cultured on fibronectin-coated dishes were used as controls for the network formation assay. Conversely, RBC depleted crude UCB cells and PB-MNCs were used as control for scRNA-seq and/or flow cytometry analysis.

### Network formation assay in Matrigel

UCB- or PB-derived adherent cells with or without OP9 pre-conditioning were seeded onto 24-well tissue culture plates coated with Matrigel matrix (Corning Inc., 354234; Corning, NY, USA) at a density of 1 × 10^5^ cells per well. DMEM (Thermo Fisher Scientific, 11885-084) with 5% UCB serum was added, and after 24 h of incubation at 37 ℃ with 5% CO_2_, cells were observed using an inverted microscope (BZ-X710, Keyence Corporation, Osaka, Japan; DMi8, Leica Microsystems, Watzler, Germany) at 10 × magnification for capillary-like formation, defined as an interconnected network structures.

For the co-culture experiments, HUVECs were labeled using a red fluorescent membrane labeling kit (Sigma-Aldrich, MINI26) according to the manufacturer’s protocol. HUVECs were mixed with 1 × 10^5^ cells of adherent cells in Matrigel at a 1:5 ratio.

### Single cell RNA sequencing

RNA-seq library construction and cDNA sequencing, including single-cell isolation, preparation of cDNA, RNA-seq library construction, cDNA sequencing, and processing, were performed by the NGS core facility of the Genome Information Research Center at Osaka University (Osaka, Japan). Analysis and graphic display derived from scRNA-seq were performed by Genble Inc.(Fukuoka, Japan). Details are described in the supplementary method 3.

### Flow cytometry

Cells were incubated for 20 min at 26 °C in the dark with fluorescent conjugated antibodies (as indicated in Table 2) and washed twice with PBS. Prior to analysis, cells were stained to removal dead cells using Cellstain-DAPI solution (1:500, Dojindo, 340-07971; Kumamoto, Japan). After staining, the cells were resuspended in 500 μL of FACS buffer for analysis using FACSAriaIII (BD Biosciences). Flow cytometric data analysis was performed using the FACSDiva software (BD Biosciences). A minimum of 20,000 events were recorded for each sample. Non-stained cells were used as control samples to determine appropriate settings for data analysis.

**Table 2:** Fluorescent conjugated antibodies used in flow cytometry analysis.

Antibody	Dilution	Catalog number
APC anti-human CD80 antibody	1:20	375404
Alexa Fluor 488 anti-human CD206 antibody	1:20	321114

All antibodies were manufactured by BioLegend.

### Animal model of permanent focal cerebral infarction by surgical ligation of the middle cerebral artery (MCAO) and administration of OP9 pre-conditioned UCB

The experimental procedure was approved by the Animal Care Committee of Hyogo College of Medicine (approval number: 19-040) and performed following the ARRIVE guidelines and the ‘Guide for the Care and Use of Laboratory Animals’ published by the National Academy of Science of the USA. Seven to nine-week-old male CB-17/Icr-+/+Jcl mice (CLEA Japan Inc., Tokyo, Japan) were housed in a temperature (22–24 °C) and humidity (55%) controlled room under a 12/12 light–dark schedule. The animals had free access to water and standard pellet chow ad libitum.

Mice were randomly assigned to three groups as follows: the UCB + OP9 group (MCAO followed by administration of OP9 pre-conditioned UCB; n = 11); the control group (the vehicle control group administered lactated Ringer’s solution alone after MCAO; n = 11), and the sham surgery group (n = 12). Two weeks after surgery, mice in the UCB + OP9 group received 100 μL of OP9 pre-conditioned UCB (2.0 × 10^7^ cells/kg) and mice in the control group received the same amount of lactated Ringer’s solution via the carotid vein under direct view.

Permanent focal cerebral infarction was induced by MCAO as described previously^40^. Briefly, under general anesthesia with 2% isoflurane (FUJIFILM Wako Pure Chemical Corporation, 099-06571; Osaka, Japan), a skin between the left eye and left ear, was incised. After removing the left zygoma using a dental drill under an operating microscope, a 1.5 mm diameter bone window was created on the surface of the skull. Finally, the proximal portion of the middle cerebral artery was exposed near the skull base and cut down just distal to the olfactory tract. This MCAO model used in the current study is known to have a clearly demarcated reproducible stroke area even in the chronic period (i.e., 14 days after MCAO induction)^66^.

Immunosuppressants were not administered because UCB is known to have low immunogenicity, and several studies have reported engraftment after xenotransplantation without immunosuppressants^20,67^.

### Fluorescent immunohistochemistry

Three months after cell transplantation, five mice in the UCB + OP9 group and five in the control group were perfused transcardially with PBS and 4% paraformaldehyde (PFA) after general anesthesia using isoflurane (FUJIFILM Wako Pure Chemical Corporation). The brains were removed and fixed overnight in 4%PFA, dehydrated in 30% sucrose, frozen at − 80 °C, and sliced into 10 μm coronal sections using a cryostat (NX70; PHC, Tokyo, Japan). Frozen sections were washed three times with PBS for 3 min each, blocked with Blocking One Histo (Nacalai Tesque, 06349-64; Kyoto, Japan), permeabilized with 1% Triton X-100 in PBS for 10 min, and washed 2 times with PBS for 5 min each. The sections were then incubated overnight at 4 °C with an anti-CD31 antibody (1:50; BioLegend, 160,202; San Diego, CA), and an anti-vWF antibody (1:50; Cell Signaling Technologies, 65,707; Danvers, MA). Alexa Fluor 488- and 647-conjugated antibodies (Thermo Fisher Scientific, SA5-10327; Cell Signaling Technologies, 4416) were used as secondary antibodies for an anti-CD31 and anti-vWF antibodies, respectively and incubated at room temperature for 2 h. Nuclei were counterstained with DAPI (SeraCare, 71-03-01; Milford, IA, USA). CD31/vWF double-positive cells in the peri-infarct lesion were recognized as endothelial cells forming microvessels^62,68–70^. Histological images were captured using a fluorescence microscope BZ-X710 (Keyence Corporation; Osaka, Japan) at 400 × magnification to measure the CD31/vWF double-positive area. Fifteen fields per group (three non-overlapping random visual fields per coronal section and one coronal section per mouse; n = 5 in each group) were analyzed. The area measurement and cell count of these cells were automatically performed in each captured image using the hybrid cell count software BZ-X analyzer in each visual field (Keyence Corporation).

### Behavioral tasks

One month after cell transplantation, behavioral tasks were performed (as indicated in the supplementary method) to assess the functional deficits and recovery of the animals after MCAO [the UCB + OP9 group (n = 11), the control group (n = 11), and the sham surgery group (n = 12)]. Based on our previous study using the same model and behavioral tasks^71^, a pre-hoc power analysis determined that a sample size of ten in each group is sufficient to have an 80% power to detect a between-subject difference among three groups in repeatedly measured ANOVA for the open field, the Y-maze, and the passive avoidance learning test. Behavioral tasks were conducted by independent experimenters, blinded to the experimental groups. Abnormal hyperactivity is known to be observed in mouse models after focal cerebral infarction and last up to 2–3 months^72,73^, which is caused by abnormal anxiety and impairment in habituation after repeated exposure due to memory disturbance^74^. In our models, these behavioral abnormalities due to MCAO can be evaluated using travel distance in the open field test^39^. Muscular strength and exercise tolerance were measured by latency to fall in the wire hang test. Spatial working memory and fear conditioned emotional memory were assessed using the Y-maze^75^ and passive avoidance learning tasks^76^, respectively. Finally, a loss of motivation and exercise tolerance were evaluated using the forced swimming test^77^. We previously confirmed the effect of MCAO induction on the scores of the Y-maze and passive avoidance learning tasks in the chronic phase (i.e. 1–2 months after MCAO induction)^71^.

### Statistical analysis

All data are expressed as mean ± standard error of the mean (SEM) and analyzed using JMP ver. 13 (SAS Institute Inc., Cary, NC, USA). Differences between two groups were analyzed using an unpaired two-tailed t-test, whereas analysis in more than two groups or in repeatedly measured values were performed using post-hoc Dunnett’s test when one-way ANOVA or rmANOVA showed statistical significance. When performing Dunnett’s test, the control group and the initial session were set as the control for the comparisons between groups and for the analysis of within-subject session-by-session differences, respectively. The statistical significance was set at *p* < 0.05.

## Supplementary Information

Supplementary Information 1.

Supplementary Information 2.

Supplementary Information 3.

Supplementary Information 4.

## Data Availability

The authors confirm that all data underlying the findings are fully available without restriction. The datasets used and/or analyzed during the current study are available from the corresponding author on reasonable request.

## Accession codes

The scRNA-seq data were deposited on NCBI GEO with the accession number GSE207249.
