# Supplementary material for: Enhanced angiogenic properties of umbilical cord blood primed by OP9 stromal cells ameliorates neurological deficits in cerebral infarction mouse model
Source: Sci Rep. 2023 Jan 6;13:262. doi: 10.1038/s41598-023-27424-7 (PMC9822952; doi:10.1038/s41598-023-27424-7)
Supplement: Supplementary file 1 — Supplementary Information 1. [file 41598_2023_27424_MOESM1_ESM.pdf]

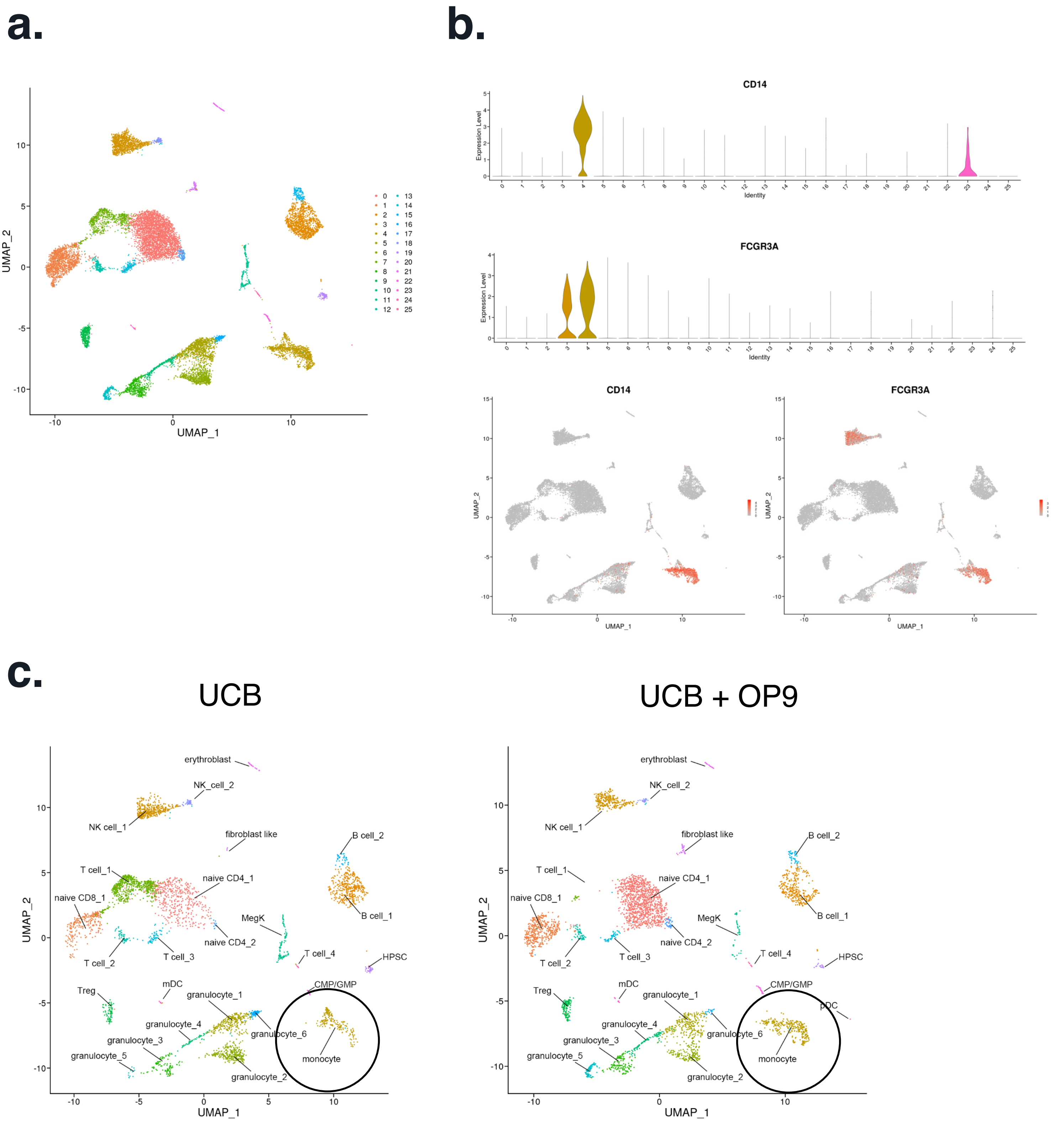

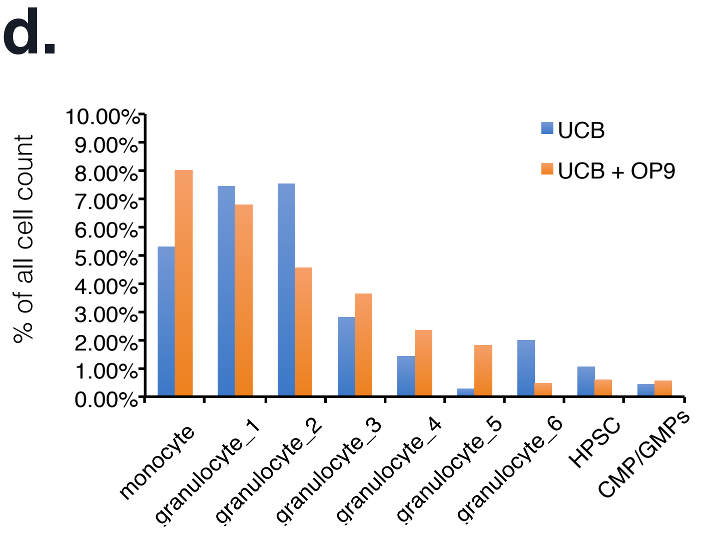

Supplementary Figure 1. t-distributed stochastic neighbor embedding (tSNE) clustering for OP9 pre-conditioned UCB and crude UCB cells

Comparison of the cell populations obtained from OP9 pre-conditioned UCB (UCB + OP9) and crude UCB (UCB). (a) Unsupervised clustering was performed using t-distributed stochastic neighbor embedding (tSNE) to organize all UCB cells into transcriptionally distinct clusters. (b) The gene expression distribution map and violin plot showed expression profiles of CD14 and CGR3A, used as monocytic marker genes. Cells expressing monocytic marker genes were highly accumulated in cluster 4 among all 25 clusters. (c) Cell types identified by the differentially expressed marker genes. (d) The ratio of cell counts in cluster_4 [mainly composed of monocytes], cluster_5, 6, 8, 10, 13 and 16 [granulocytes], cluster_19 [hematopoietic stem cells (HPSCs)], and cluster_22 [granulocyte-macrophage progenitors (CMP/GMPs)] to all cell counts in UCB and UCB + OP9 cells.
