# Supplementary material for: Enhanced angiogenic properties of umbilical cord blood primed by OP9 stromal cells ameliorates neurological deficits in cerebral infarction mouse model
Source: Sci Rep. 2023 Jan 6;13:262. doi: 10.1038/s41598-023-27424-7 (PMC9822952; doi:10.1038/s41598-023-27424-7)
Supplement: Supplementary file 2 — Supplementary Information 2. [file 41598_2023_27424_MOESM2_ESM.pdf]

UCB + OP9

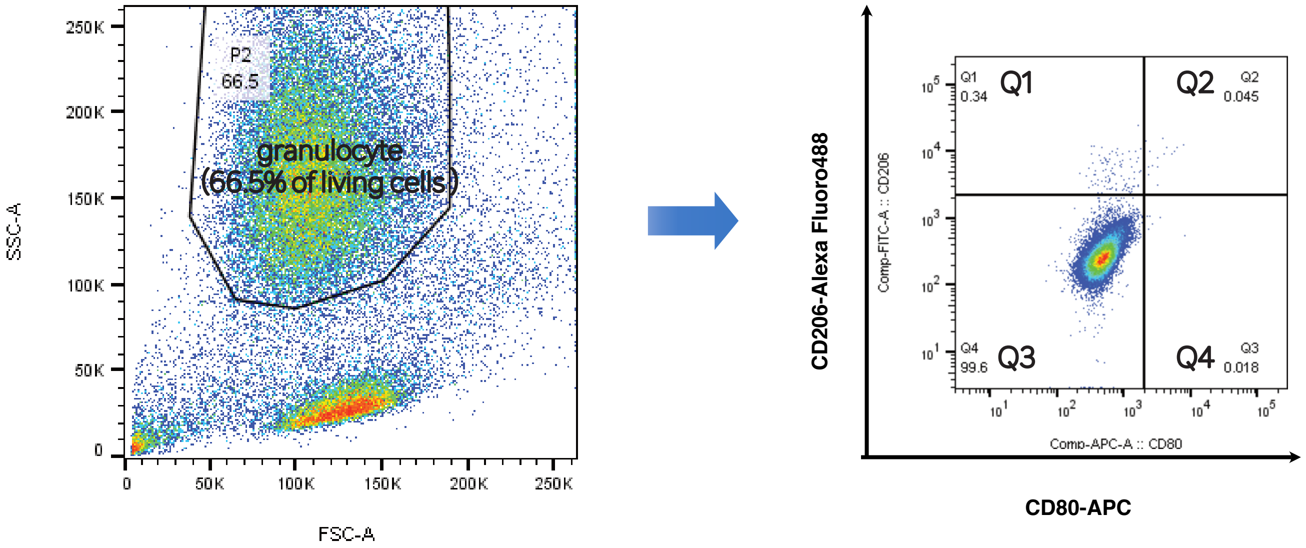

Supplementary Figure 2. Flow cytometry analysis of OP9 pre-conditioned UCB cells.
Flow cytometry analysis of UCB + OP9. Cells in granulocytic fraction (P2 population) were gated based on forward angle light scatter (FSC) and side angle light scatter (SSC) characteristics. Then, CD80 and CD206 were used to sort N1 and N2 type cells into Q4 and Q1 quadrants, respectively.
