# Supplementary material for: Enhanced angiogenic properties of umbilical cord blood primed by OP9 stromal cells ameliorates neurological deficits in cerebral infarction mouse model
Source: Sci Rep. 2023 Jan 6;13:262. doi: 10.1038/s41598-023-27424-7 (PMC9822952; doi:10.1038/s41598-023-27424-7)
Supplement: Supplementary file 3 — Supplementary Information 3. [file 41598_2023_27424_MOESM3_ESM.docx]

**Supplementary method: Detailed protocols for scRNA-seq and behavioral tests**

**1. Detailed protocols for scRNA-seq**

***1.1 scRNA-seq library construction, sequencing and processing***

RNA-seq library construction and cDNA sequencing, including single-cell isolation, preparation of cDNA, RNA-seq library construction, cDNA sequencing, and processing, were performed by the NGS core facility of the Genome Information Research Center at Osaka University (Osaka, Japan). Single-cell suspensions were processed using the 10x Genomics Chromium Controller (10x Genomics, Pleasanton, CA) following the protocol outlined in the Chromium Single Cell 3' Reagent Kits User Guide. The Chromium Next GEM Single Cell 3' Kit v3.1 (PN-1000269), Chromium Next GEM Chip G Single Cell Kit (PN-1000127), and Dual Index Kit TT Set A (PN-1000215) were used during the process.

Briefly, UCB cells with or without OP9 pre-conditioning underwent a single-cell capture procedure. Single-cell suspensions containing approximately 16,500 live cells per sample were loaded into a chromium controller to generate 10,000 single-cell gel-bead emulsions for library preparation and sequencing. Oil droplets of encapsulated single cells and barcoded beads (GEMs) were subsequently reverse-transcribed in a Veriti Thermal Cycler (Thermo Fisher Scientific), resulting in cDNA tagged with a cell barcode and a unique molecular index (UMI). Then, cDNA was amplified to generate single-cell libraries, according to the manufacturer’s protocol. Quantification was performed using an Agilent Bioanalyzer High Sensitivity DNA assay (Agilent, High-Sensitivity DNA Kit, 5067-4626; Santa Clara, CA, USA). The amplified cDNA was enzymatically fragmented, end-repaired, and polyA-tagged. Cleanup/size selection was performed on amplified cDNA using SPRIselect magnetic beads (Beckman-Coulter, SPRIselect, B23317; Brea, CA, USA). Next, Illumina sequencing adapters were ligated to size-selected fragments and cleaned using SPRIselect magnetic beads. Finally, the sample indices were selected and amplified, followed by double-sided size selection using SPRIselect magnetic beads. The quality of the final library was assessed using an Agilent Bioanalyzer High-Sensitivity DNA assay. Samples were then sequenced on an Illumina NovaSeq 6000 paired-end mode (read1:28bp; read2:91bp). The resulting raw reads were subjected to demultiplexing, alignment, barcode counting, UMI counting, and filtering using Cell Ranger v.6.0.0 (10x Genomics). The human genome (GRCh38) was used as a reference to align the reads.

***1.2 Aanalysis and graphic display of single cell RNA-seq data***

Analysis and graphic display derived from scRNA-seq were performed by Genble Inc.(Fukuoka, Japan). The following analysis was performed in R (version 4.0.5), using the Seurat package (version 4.0.5). Briefly, cells that had more than 15% of mitochondrial-associated genes among their expressed genes, cells with <200 or >4,000 genes, and cells with more than 25,000 UMI counts were considered outliers and filtered out. UMI counts were log-normalized (scale factor = 10,000). We regressed the S scores, G2/M scores, and the percentage of mitochondria-associated genes during data scaling.

Dimensionality reduction was performed using principal component analysis, and the first 75 principal components were used to identify distinct cell populations. We performed cell clustering using the shared nearest neighbors method and visualized it in two dimensions using the uniform manifold approximation and projection. The top preferentially expressed genes in several clusters included multiple known markers of particular cell types, where we identified the monocytic population.^1,2^ Differentially expressed genes (DEGs) between the UCB and UCB + OP9 groups in the monocytic cluster were analyzed using MAST in the Seurat package.^3^ The significance threshold was set as |log2(fold change)| ≧ 0.5 and adjusted *p* value < 0.001. Gene ontology enrichment analysis was performed using clusterProfiler R package.^4^ Significantly enriched GO terms were identified based on the hypergeometric test from each input list of DEGs, and adjusted *p* values were derived from the Benjamini & Hochberg (BH) method. The top 15 enriched GO terms were identified according to the –log_10_ (*P*-value).

The pheatmap R package was used to perform k-means clustering with Pearson correlation as the distance metric and to construct heatmaps of typical M1 and M2 marker gene expressions. M1 and M2 signature genes were extracted from the Gene Expression Omnibus (GEO) dataset GSE5099, Martinez et al., and Medina et al.^5,6^

**2. Detailed protocols for behavioral tests**

***2.1. The open field test***

Spontaneous locomotor activity was examined using a cubic open field box (30 × 30 × 30 cm) made from transparent acrylic plates (30 × 30 × 30 cm). The apparatus was illuminated by indirect lighting with approximately 150 lux brightness on the floor of the open field box. Each mouse was placed at the center of the box and could freely explore the arena for 10 min per day for three consecutive days. The behaviors of the mice were recorded with a digital video camera placed above the open field box. The distance traveled in the open-field box was calculated using a computerized video-based tracking system (Be-Chase ver.2021; ISONIX Co. Ltd., Kobe, Japan).

***2.2. The wire hang test***

A grid plate was made of a square metal flame (36 × 30 cm) and rods (3 mm in diameter and placed at 10 mm intervals). First, each mouse was placed on the grid plate for 10 sec for acclimatization. The testing plate was then gently inverted and placed on the top of a cubic open-topped glass box (30 × 30 × 30 cm). Latency to fall was measured, with a maximum trial time of 180 s. This trial was repeated three times with an interval of 10 min for each condition.

***2.3. Y-maze task***

A Y-shaped maze has 3 arm runways (length: 40 cm, width: 3 cm, height: 20 cm) diverging at 120° angles from a central hub. The Y-shaped maze was set on a pedestal (height: 30 cm) and enclosed by 120cm high white walls. The maze was illuminated by indirect lighting with 250 lux brightness on the maze floor. Each mouse was placed onto the central area and allowed to explore freely through the maze for 5 min. The behaviors of the mice were recorded with a digital video camera placed above the maze. A mouse was considered to enter an runway when all 4 paws entered in the runway, and the sequence of arm entries was manually defined. An alternation was defined as an entry into all 3 different arms in sequence on consecutive occasions (e.g., ABC, CBA, BAC). The maximum alternation was calculated as the total number of arm entries minus 2, and the percentage of alternation was calculated as (actual alternation / maximum alternation) × 100.

***2.4. Passive avoidance learning test***

A step-through-type passive avoidance learning apparatus (MPB-M020; Melquest Ltd, Toyama, Japan) was used. The apparatus was composed of a light (10 × 10 × 15 cm) and a dark (20 × 20 × 15 cm) compartments separated by a guillotine door. The light compartment was illuminated with a white LED light (400 lux), whereas the other remained dark (20 lux).

In the conditioning trial, mice were individually placed in the light compartment and allowed to explore freely. Ten seconds later, the guillotine door was opened to allow the mouse to enter the dark compartment. When the mouse moved into the dark compartment, the guillotine door was closed. 10 seconds later, an electrical foot shock (0.2 mA, 5 s) was delivered through the grid floor.

Twenty four hours (test1) and forty eight hours (test2) later, the retention test trials without any shock were conducted. The subjected mouse was placed in the light compartment and the duration to enter the dark compartment was recorded up to 300 seconds.

***2.5. Forced swimming test***

The forced swimming test was conducted using a small cylindrical tank (inside diameter: 18 cm, depth: 29 cm) containing water to a depth of 15 cm. The temperature of the water was maintained at 24 ± 1℃. A ceiling fluorescent light provided indirect lighting with approximately 150 lux brightness at the water surface.

Mice were individually placed in the tank for 6 min. The behaviors of the mice were recorded with a digital video camera placed above the tank. The computerized video-based tracking system (Be-Chase ver.2021; ISONIX Co. Ltd., Kobe, Japan) calculated the total amount of immobility by measuring the duration that the mouse swam below the specified threshold velocity of 3.0 cm/s.

1 Tirosh, I. *et al.* Dissecting the multicellular ecosystem of metastatic melanoma by single-cell RNA-seq. *Science* **352**, 189-196, doi:10.1126/science.aad0501 (2016).

2 Nestorowa, S. *et al.* A single-cell resolution map of mouse hematopoietic stem and progenitor cell differentiation. *Blood* **128**, e20-31, doi:10.1182/blood-2016-05-716480 (2016).

3 Finak, G. *et al.* MAST: a flexible statistical framework for assessing transcriptional changes and characterizing heterogeneity in single-cell RNA sequencing data. *Genome Biol.* **16**, 278, doi:10.1186/s13059-015-0844-5 (2015).

4 Yu, G., Wang, L. G., Han, Y. & He, Q. Y. clusterProfiler: an R package for comparing biological themes among gene clusters. *OMICS* **16**, 284-287, doi:10.1089/omi.2011.0118 (2012).

5 Martinez, F. O., Gordon, S., Locati, M. & Mantovani, A. Transcriptional profiling of the human monocyte-to-macrophage differentiation and polarization: new molecules and patterns of gene expression. *J. Immunol.* **177**, 7303-7311, doi:10.4049/jimmunol.177.10.7303 (2006).

6 Medina, R. J. *et al.* Myeloid angiogenic cells act as alternative M2 macrophages and modulate angiogenesis through interleukin-8. *Mol. Med.* **17**, 1045-1055, doi:10.2119/molmed.2011.00129 (2011).
